# High concentrations of LH cause virilization in a postmenopausal woman

**DOI:** 10.1002/ccr3.803

**Published:** 2017-01-27

**Authors:** Iram Ahmad, Bradley D. Anawalt

**Affiliations:** ^1^ Division of Metabolism, Endocrinology and NutritionDepartment of MedicineUniversity of WashingtonSeattleWashingtonUSA; ^2^ Division of General Internal MedicineDepartment of MedicineUniversity of WashingtonSeattleWashingtonUSA

**Keywords:** Adrenal tumors, GnRH analog use, high testosterone levels, hirsutism, postmenopausal virilization

## Abstract

Some testosterone‐producing adrenal tumors can be successfully treated with long‐acting GnRH analogs and adrenal venous sampling can be useful to detect rare ectopic sex steroid‐producing tumors or confirm bilateral adrenal androgen hyperproduction in female hyperandrogenism.

## Patient Presentation

A 62‐year‐old woman presented with a 2‐year history of increased facial hair, male pattern baldness, and clitoromegaly. Past medical history was significant for osteopenia and migraine headaches. She had menarche at age 12, had two biological children with no problems with fertility, and underwent natural menopause at age 51. She reported that her husband and other male family members were not taking testosterone or androgen precursors. Initial assessment revealed a markedly elevated total testosterone concentration in 2012, and patient was started on spironolactone. She began taking over‐the‐counter Estroven™ for 8 months, and then was switched to transdermal estradiol patch 0.05 mg/day for approximately 2 months. She reported no improvement with systemic estrogen therapy.

A 2013 CT scan revealed possible left adrenal thickening with tiny punctate calcifications consistent with old infection/inflammation or hemorrhage. A transvaginal ultrasound showed a 5.5‐cm fibroid displacing the endometrial stripe and normal ovaries. The patient was diagnosed with ovarian hyperthecosis and underwent total vaginal hysterectomy with bilateral salpingo‐oophorectomy in March 2014, a year prior to her presentation to us.

Laboratory work performed by her primary care physician showed unchanged serum testosterone levels after total vaginal hysterectomy with bilateral salpingo‐oophorectomy (Table 1).

**Table 1:** Pre‐ and post bilateral oophorectomy hormone concentrations

	11/15/13	11/20/13	1/8/14	5/26/14
Total T (2–45 ng/dL)	**337**	**281**	**230**	***288***
Free T (0.1–6.4 pg/mL)		**41.2**	**42.4**	***55.4***
DHEA (<1185 mcg/dL)	117			
DHEA‐S (24–278 mcg/dL)	92			
Estradiol (5–38 pg/mL)			23	
FSH (0–14 mIU/mL)	**86.5**			
LH (0–14 mIU/mL)	**42.7**			
17‐hydroxyprogesterone (<51 ng/dL)			26	
Prolactin (<20 ng/mL)			8	
ACTH (6–50 pg/dL)		9		

Abnormal results in bold; post hysterectomy laboratories in bold italics.

At this point, the patient was referred to our medical center for further evaluation. Repeat laboratory tests by our institution yielded results similar to those obtained previously. Repeat CT scan of the abdomen and pelvis performed at our institution in August 2014 revealed a 7‐mm left adrenal adenoma (20 Hounsfield units) and no evidence of pelvic ovarian remnants. We compared our CT imaging with the 2013 CT and noted that the adrenal nodule had been subtle, but visible on the 2013 CT; the adrenal nodule was unchanged in size and shape.

We recommended adrenal venous sampling with cosyntropin stimulation to confirm that the hyperandrogenism was adrenal in origin and to attempt to determine whether the left adrenal nodule was the source of the excess testosterone (Table 2).

**Table 2:** Venous serum hormone concentrations before and after cosyntropin administration

	17‐hydroxyprogesterone (ng/dL)	Total Testosterone (T) (ng/dL)	Cortisol (mcg/dL) (c)	Poststimulation Testosterone:Cortisol Ratio
Left adrenal	96→28,400	118→572	28.9→1464	0.39
Right adrenal	49→17,300	136→343	16.1→817.5	0.41
Peripheral	<40→58	192→341	6.0→15.6	

Data presented as prestimulation with cosyntropin → Poststimulation with cosyntropin.

These results show that (i) the adrenals were the source of the androgen excess, (ii) there was no lateralization, and (iii) the androgen excess was ACTH responsive.

To further elucidate whether the adrenal production of androgen was regulated by ACTH, the patient underwent 1‐ and 3‐day dexamethasone suppression, with 1 mg of dexamethasone each night, at her clinic in Idaho (Table 3).

**Table 3:** Results of low‐ and high‐dose dexamethasone suppression tests

	After 1 dose	After 3 doses
Cortisol (mcg/dL)	1.2	1.2
Total Testosterone (ng/dL)	233	131

Testosterone partially suppressed with dexamethasone, which confirmed that the androgen excess was partly regulated by ACTH.

To determine whether the hyperandrogenism was LH responsive, we measured serum LH and testosterone concentrations with a single dose of leuprolide 3.75 mg, a potent long‐acting gonadotropin‐releasing hormone (GnRH) analog. Her serum LH suppressed from 42.7 IU/L to 5.5 IU/L, and her serum total testosterone concentration nearly normalized (58 ng/dL). Serial testosterone and LH levels were tested after initiation of leuprolide, 3.75 mg every eight to 10 weeks (Table 4).

**Table 4:** Continued suppression of LH and testosterone with leuprolide 3.75 mg every 8–10 weeks

	Weeks after initiation of leuprolide			
	15	18	30	55
LH (mIU/mL)	4.0	34	39.9	1.5
Total Testosterone (ng/dL)	43	63	104	7

## Discussion

The source of hyperandrogenism in a postmenopausal female provides several diagnostic challenges. During menopause, while estrogen production diminishes, testosterone production remains stable, which can cause an imbalance in the estrogen to testosterone ratio and cause hyperandrogenic symptoms 1.

Clinical history is essential to delineate clinical course of the hyperandrogenism. It is important to inquire about exposure to androgens, androgen precursors, and androgenic anabolic steroids. Manifestations of some forms of hyperandrogenism, such as congenital adrenal hyperplasia or polycystic ovarian syndrome, present in puberty, but the androgenic symptoms (alopecia and hirsutism) may become more pronounced with the onset of menopause and circulating decline in estrogens 2. In this patient, the absence of premenopausal symptoms made it unlikely that her symptoms were due to an aggravation of a previously undiagnosed form of hyperandrogenism 3.

Potential causes of androgen excess in this case included the pituitary gland, ovaries and the adrenal glands. In regard to her pituitary, a gonadotrophinoma is extremely unlikely. Gonadotrophinomas that secrete significant amounts of bioactive LH or FSH are extremely rare, and it would be highly unusual for a gonadotrophinoma to increase immunoreactive, let alone bioactive, serum LH and FSH concentrations to the extent seen in this postmenopausal woman 4. Gonadotrophinomas of clinical significance are generally large, and patients have loss of vision, hyperprolactinemia and hypopituitarism 5. This patient had a low‐normal serum prolactin and normal T4 and TSH concentrations. Additionally, in a gonadotrophinoma, autonomous secretion of gonadotropins would be unlikely to be suppressed by a GnRH analog. Based on her laboratory profile, she had to be either producing testosterone or taking testosterone or a testosterone precursor. Her markedly elevated gonadotropins are inconsistent with exogenous testosterone or testosterone precursor as an explanation for her very high serum testosterone concentrations.

As her initial imaging was interpreted as showing no ovarian or adrenal tumor, ovarian hyperthecosis or an occult, small androgen‐secreting Leydig cell tumor that is not easily visualized was suspected 6. However, bilateral oophorectomy had no effect on her serum total testosterone concentration.

Based on the onset of her symptoms with menopause, we hypothesized that her persistent hyperandrogenism was due to a LH‐stimulated adrenal source. Adrenal venous sampling is generally not useful in the evaluation of female hyperandrogenism, but in this case, adrenal venous sampling was helpful to exclude an ovarian source (from a small remnant not removed during surgery) and confirm that the left adrenal tumor was not the source of hyperandrogenism. Dynamic testing with cosyntropin stimulation and dexamethasone suppression further confirmed the adrenal source of hyperandrogenism. Diagnostic and therapeutic use of a potent GnRH analog demonstrated that the hyperandrogenism was due to LH stimulation of adrenal androgen production.

Low levels of LH receptors are found on nontumorous adrenal glands, but their role outside of the context of adrenal carcinomas is unclear. The levels of these receptors can increase in the adrenal cortex after exposure to chronically elevated gonadotropins. Bernichtein, et al., speculate that high serum LH concentrations and adrenal LH receptor expression are not generally sufficient to stimulate adrenal‐mediated hyperandrogenism; additional alterations in transcription factors are necessary to enhance LH receptor expression and trigger adrenal hyperandrogenism 7.

It has been documented in the literature that suppression of hyperandrogenemia with long‐acting GnRH analogs is highly suggestive of an ovarian etiology 8, 9, but in this patient, such an interpretation would have been inaccurate.

In conclusion, this woman developed significant hyperandrogenism likely due to the normal menopausal rise of serum LH concentrations, thereby stimulating LH adrenal receptors and adrenal testosterone production. Her presentation illustrates that suppression of serum testosterone with a GnRH analog is not diagnostic of ovarian source of hyperandrogenism, which has been described previously 10. Although adrenal venous sampling is not useful in the routine evaluation of female hyperandrogenism, it can be useful in detecting an adrenal from an ectopic, nonadrenal source of androgen hyperproduction or, as in this case, to confirm bilateral adrenal androgen hyperproduction.

## Conflict of Interest

None declared.

## Authorship

IA: wrote and edited the manuscript and subsequent revisions, interpreted the data, contributed to intellectual content, and takes responsibility for the accuracy of the case presentation. BA: conceived and oversaw the implementation of the treatment plan, reviewed and edited subsequent revisions of the manuscript, and takes responsibility for the accuracy of the case presentation. All authors had full access to all data.
